# Sciatic neurectomy-related cortical bone loss exhibits delayed onset yet stabilises more rapidly than trabecular bone

**DOI:** 10.1016/j.bonr.2021.101116

**Published:** 2021-08-17

**Authors:** Samuel Monzem, Behzad Javaheri, Roberto Lopes de Souza, Andrew Anthony Pitsillides

**Affiliations:** a Skeletal Biology Group, Comparative Biomedical Sciences, Royal Veterinary College, Royal College Street, NW1 0TU London, United Kingdom; b Federal University of Mato Grosso, Veterinary College, Av. Fernando Correa da Costa, n. 2367, 78060-900 Cuiabá-Mato Grosso, Brazil

**Keywords:** Bone resorption, Bone loss trajectory, Bone mass and shape, Cortical and trabecular bone, Disuse

## Abstract

Disuse osteoporosis occurs after extended periods of bed rest or nerve damage leading to increased risk of fracture. It remains to be established, however, whether the trajectory of bone loss is equivalent in bone's cortical and trabecular compartments following long-term periods of reduced loading. Herein, we evaluate sciatic neurectomy-related cortical and trabecular bone loss in the tibia by microCT. The right hind limb of seventeen 12 week-old female mice was subjected to sciatic neurectomy (right, SN; left, contralateral internal control) and the animals were sacrificed in four groups (*n* = 3-5/group) at 5, 35, 65 and 95 days thereafter. Cortical bone mass, geometry and mineral density were evaluated along almost the entire tibial length and trabecular bone was examined at the proximal metaphysis. We found that trabecular bone volume (BV/TV) and number were decreased within 5 days, with a trajectory of loss that only plateaued after 65 days post-SN. In contrast, decreases in cortical thickness, cross-sectional area, second moment of inertia along minor and major axes and predicted resistance to torsion were unmodified during the early 5 day period, attaining significance only after 35 days post-SN and, thereafter showed no further deterioration. Only cortical ellipticity and periosteal enclosed area, continued to change in the SN limbs (vs. contralateral) between 35 and 95 days along the tibia length. On the other hand, cortical tissue mineral density was unmodified by SN at any time point. These data indicate that SN-related cortical bone loss extends along almost the entire tibia, exhibits delayed onset and yet stabilises its architecture more rapidly than trabecular bone. These data suggest that the cortical and trabecular compartments behave as distinct modules in response to SN even within an individual bone.

## Highlights

• Responses to sciatic neurectomy (SN) in tibial trabecular bone are very protracted.
• Responses to SN in the cortices have delayed onset but stabilize more rapidly.
• Trabecular and cortical bone behave modularly in response to SN.

## 1 Introduction

Bone loss occurs in humans during bed rest, after a stroke or a spinal cord injury and in astronauts during spaceflight (de Brito et al., 2013; Grimm et al., 2016; Dauty et al., 2000). These observations are consistent with the triggering of bone's mechanostat by the reduction of mechanical loading to increase resorption and diminish formation (Weinreb et al., 1989; Frost, 1987). The consequences are the deterioration in both cortical and trabecular bone mass and architecture (Traon et al., 2007; Bikle and Halloran, 1999). It remains to be established, however, if interventions that aim to mitigate against these consequences should preferential target the trabecular or cortical bone compartment based on any differences in their relative sensitivity to, or trajectory of bone loss associated with the reduced loading.

These deteriorations in bone are known to start rapidly in humans that have suffered spinal cord injury and to continue at a low level for more than one year; reports vary regarding the duration of time taken before this bone loss plateaus, varying from 1.2 to 3.6 years among individual patients (Haider et al., 2018). The scale of the bone loss in these spinal cord injury patients can also be dramatic, with the tibia losing 70% of its mass and 41% of its mineral density (Dauty et al., 2000; Haider et al., 2018). The scale of the diminution of mineral content and bone stiffness in response to the associated reductions in loading may also show local variation even within an individual bone, where decreases appear greater in trabecular-rich epiphyseal regions than in the cortical compartment at the diaphysis (Dauty et al., 2000; Haider et al., 2018). Our understanding of the basis for these temporal-spatial variations in bone loss as well as their magnitude remains, however, incomplete.

Animal models of diminished skeletal *loading* are used extensively to mimic the bone loss that occurs in response to microgravity, bed rest or lack of sufficient loading stimulus (Otzel et al., 2019; Jiang et al., 2006). These models use rats, mice or chicks and include hindlimb elevation by tail suspension, where the hind limbs are able to move but do not carry weight, immobilization through the application of botulinum toxin A to the calf muscles and bone loss evoked by surgical resection of the sciatic nerve or spinal cord in which weight-bearing is significantly compromised (Weinreb et al., 1989; Otzel et al., 2019; De Souza et al., 2017; Biewener and Bertram, 1994; Ausk et al., 2017; Yang et al., 2019; Lodberg et al., 2015). Studies using these models have disclosed that cortical and trabecular bone do not necessarily behave identically in response to the imposition of reduced loading. Predominant evidence indeed aligns with the notion that bone with a trabecular organisation is subject to more marked and more rapid deterioration in architecture, is more sensitive to reduced loading than cortical tissue and, thus, more realistically reproduces the characteristics of human bone changes due to spinal cord injury or sciatic neurectomy (SN) (Otzel et al., 2019; Piet et al., 2019).

This view is further strengthened by data from a rat model of spinal cord injury, where cortical and trabecular deterioration is more marked than in response to bilateral SN (Jiang et al., 2006), and where deficits in trabecular bone architecture are very rapid compared to the only gradual bone loss observed in the cortical compartment. Similar studies have reported >50% decreases in trabecular bone volume within only 14 days (Otzel et al., 2019), yet as little as a 10% decrease in cortical bone mass between the tibia's proximal end and its distal tibiofibular junction after 19 days (Piet et al., 2019) in a model of reduced loading evoked by SN.

It has been highlighted that the osteoclastic bone resorption induced as a result of reduced loading due to spinal cord injury or SN also leads to shifts in bone organ shape, with acquisition of reduced curvature levels (development of straighter shaped bones) with modified predicted distributions of habitual strain on their surfaces as well as poorer mechanical properties (Otzel et al., 2019; Jiang et al., 2006; Biewener and Bertram, 1994; Bertram and Biewener, 1988). These changes in bone cell behaviour induced in these models of reduced loading can be very rapid; muscle paralysis can generate an inflammatory response that engenders very rapid increases in both the number and size of osteoclasts, prompting fast changes in resorption and consequent disruption of bone architecture, mass and shape (Weinreb et al., 1989; Biewener and Bertram, 1994; Ausk et al., 2017). The number of osteoclasts actively resorbing bone increases dramatically within the early stages (72 h) after SN but indeed diminishes to control levels at later stage (Weinreb et al., 1989). The osteoblast is also highly sensitive to the effects of reduced mechanical loading and exhibits a reduced function within brief periods of disuse imposed by SN (Weinreb et al., 1991).

Previous elegant studies have reported upon the distribution and timing of bone loss under a range of reduced loading conditions (Otzel et al., 2019; Jiang et al., 2006; De Souza et al., 2017; Kingery et al., 2003). Nonetheless, the trajectory and relative scale of the bone loss engendered by SN in the trabecular compartment and the entire cortex of a single bone has not previously been fully described. This study will explore whether trabecular or cortical compartments exhibit conserved or modular behaviour in terms of the scale and timing of the response to imposed SN, by assessing changes in metaphyseal trabecular regions and along the cortices of the entire tibia after 5, 35, 65 and 95 days.

## 2 Materials and methods

### 2.1 Animal

Seventeen, 12-week-old female C57BL/J6 mice were housed in groups of four/five in polypropylene cages and subjected to 12 h light/dark cycle, with room temperature maintained between 19 and 23 °C, and fed ad libitum with a maintenance mice diet and water. All procedures complied with the Animals (Scientific Procedures) Act 1986, local ethics committee and were cover by licence number 70/07859.

### 2.2 Anaesthesia, surgery and groups

Each mouse was pre-medicated subcutaneously with 0.1 mg/kg buprenorphine (Vetergesic; Animalcare, York, UK) and anaesthesia was induced and maintained with isoflurane (Isoforine®-Cristália) diluted in 100% oxygen delivered by mask. Sciatic neurectomy (SN) of the right limb of each mouse was accomplished as previously described and the contralateral left limb served as an internal contralateral control (contralateral) (De Souza et al., 2017; de Souza et al., 2005). Briefly, an incision was made caudal to the right hip joint and the biceps femoris muscle elevated to expose the nerve. SN was achieved by resecting a 3-4 mm segment of the sciatic nerve posterior to the hip joint. The neurectomised mice were able to move around in the cage and gained access to food and water without difficulties. The mice were sacrificed through cervical dislocation at one of four different time points: either 5 days (*n* = 5), 35 days (*n* = 4), 65 days (n = 5) or 95 days (*n* = 3) after right SN. The tibiae, both left and right, were subsequently dissected, fixed in neutral-buffered formaldehyde for 24 h before washing and storage in 70% alcohol prior to microCT scanning.

### 2.3 Assessment of bone mass and shape changes in cortical and trabecular compartments of the tibia

To enable the effects of different durations of SN on bone mass, shape and architecture to be evaluated, left (contralateral) and right (SN) tibia of each mouse were scanned with the X-ray microcomputed tomography-Skyscan 1172 (microCT) (Skycan, Kontich, Belgium) with the following scanning set up: 0.5 mm aluminium filter, medium camera, 5 μm voxel size, tube operated at 49kv/200μa and 1600 ms exposure time. After scanning, the slices were obtained with NRecon 1.7.1.0 (Skycan, Kontich, Belgium) software.

### 2.4 Trabecular analysis

To assess bone architecture, tibiae were re-orientated in Data-Viewer 1.5 (Skycan, Kontich, Belgium) which allowed the selection of a region of interest (ROI) by CTAn software in identical orientation. These were then used to select the first slice within the secondary spongiosa, in which both lateral/medial aspects contained bone, and a distal ROI of the diaphysis, 5% of total bone length, selected for analysis. These ROIs were manually drawn and a threshold of 80 chosen to reflect true trabecular thickness in the binarized image for 3D analyses for trabecular bone volume (BV/TV), trabecular number (Tb.N), trabecular separation (Tb.Sp), trabecular thickness (Tb.Th), connectivity density (Conn.D), bone mineral density (BMD) and tissue mineral density (Tb.TMD) were also measured within the same ROI after appropriate phantom calibration in CTAn Software.

### 2.5 Cortical analysis along the tibia length

After initial measurement of the length of the entire tibia using CTAn, cortical bone mass and shape along the tibia length (15% to 85% - proximal diaphysis forward distal) was evaluated using previously published methodology (Javaheri et al., 2018). Briefly, the tibia slices were imported to Image J software to remove the fibulae and, thereafter, the Bone J plugin was used with a minimum and maximum threshold of 80 and 255 respectively, to best select bone tissue area without over-accommodating bone pores. This allowed measurement of mean cortical thickness (Ct.Th), cortical cross-sectional area (CSA) and second moment of area around major/minor axes (I_min_/I_max_), which were used to calculate predicted resistance to torsion (J; by adding I_min_ and I_max_) and cortical ellipticity (by dividing I_min_ to I_max_). The total tissue area (Tt.Ar, periosteal enclosed area) and tissue mineral density (TMD) were also measured using 2D analyses, after appropriate phantom calibration in CTAn Software (Javaheri et al., 2018).

### 2.6 Statistical analysis

Data from tibia length and trabecular bone, including BV/TV, Tb.N, Tb.Sp, Tb.Th, Conn.D, BMD and Tb.TMD were each tested for normality of variance using a Shapiro-Wilk test to verify adherence to a normal distribution and analysed through Two-way ANOVA followed by Tukey's post-test for significance. All trabecular analyses were performed using Graph Pad Prism 8.2.0, and significance was achieved with *P*-values <0.05.

Data from the tibial cortical bone, including Ct.Th, CSA, I_min_, I_max_, Tt.Ar and TMD were integrated into a single file to incorporate the position along the tibia proximo-distal length (15% - 85%). This was used in order to perform statistical analysis between the tibiae subjected to SN and the contralateral limb, and between the different times points (5, 35 65 and 95 days) using Two-way ANOVA followed by Tukey's post-test for significance. All of these analyses of cortical bone were performed using R software (version 3.4.4.4) and distinct levels of statistical significance set at *P* < 0.001, 0.001 < *P* < 0.01 and 0.01 < *P* < 0.05.

## 3 Results

### 3.1 SN-related changes in the trabecular bone compartment are both rapid in onset yet protracted

Twelve week-old mice were maintained up to an age of ~25 weeks during the 5-95 day experimental phase, which corresponds to a period of bone growth. Bone length was therefore assessed and showed that SN did not exert significant effects on longitudinal growth at any time-point (vs. contralateral). This was despite increases in tibia length in contralateral limbs between 35 and 65 days which failed however to reach significant levels across this period in limbs subjected to SN (data not shown).

**Fig. 1:**
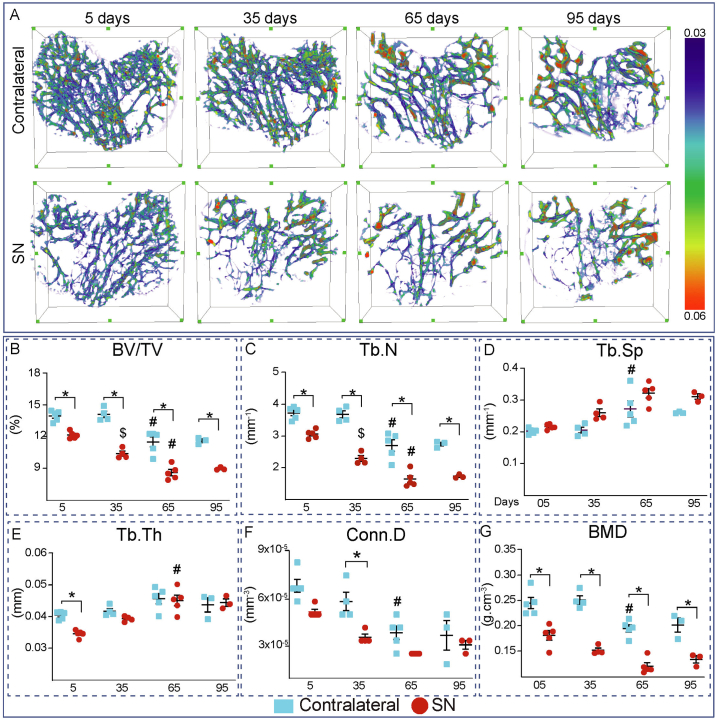
Trabecular architecture declines at 5 days and saturates at 65 days of Sciatic neurectomy. Mean ± SEM of A: Trabecular thickness heat map, B: trabecular bone volume (BV/TV), C: trabecular Number (Tb.N), D: Trabecular Separation (Tb.Sp), E: trabecular Thickness (Tb.Th), F: connectivity Density (Conn.D), G: Bone mineral density (BMD) of 12 week-old-mice left tibia (Contralateral) and right tibia (Sciatic Neurectomy-SN) after 5, 35, 65 and 95 days of SN. Statistical significance: * *p* < 0.05 when compare between groups (contralateral vs SN). $: p < 0.05 when compare 5 vs 35 days inside same group (contralateral vs contralateral or SN vs SN). #: p < 0.05 when compare 35 vs 65 days inside same group (contralateral vs contralateral or SN vs SN).

Examination across a conserved lengthwise trabecular ROI revealed modification in contralateral tibiae (Fig. 1), with an age-related decline particularly between 35 and 65 days for BV/TV, trabecular number, connectivity density and BMD (Fig. 1B, C, F and G); these were accompanied by opposing increases in trabecular separation (Fig. 1D). Examination of the effects of SN (vs. contralateral) showed a rapid and significant bone loss within the initial 5 days of SN, which progressed at later timepoints, consistent with a protracted trabecular decline which extended through to 65 days post-SN. These trabecular deficiencies were evident and significant within 5 days of SN for BV/TV, trabecular number and BMD and, remained reduced relative to contralateral limb levels at all timepoints thereafter (Fig. 1B, C and G). On the other hand, the trabecular connectivity diverged but was lower in SN than in contralateral limbs only at 35 days (Fig. 1F); no significant shifts in Tb.TMD were evident in SN limbs (vs. contralateral) at any time point.

SN-related declines in BV/TV and trabecular number (vs. contralateral) continued between both 5-35 and 35-65 days. Evidence for a plateau in SN-related bone loss was observed in the lack of any further modification in any trabecular parameters in the SN limbs between 65 and 95 days (Fig. 1B–G). As expected, trabecular separation showed opposing trends but did not attain any significant modification in response to SN (vs. contralateral) at any time-point (Fig. 1D).

Together, these data show that SN affects the trabecular compartment to produce deficiencies in architecture, particularly in BV/TV and Tb.N within 5 days that were exacerbated dramatically during both the subsequent 5-35 and 35-65 day-long periods. These SN effects were only ‘saturated’ in the final 65-95 day period when no further SN-related deteriorations in trabecular architecture were apparent. Tb.Th deviated markedly, however, from this pattern; consistent with other changes Tb.Th was significantly reduced within 5 days of SN, but intriguingly was increased between 5 and 35 days and significantly between the 35-65 day timepoints, after which no further modification was evident (Fig. 1E). Only BMD was significantly lower in the SN limbs at all timepoints without showing any further significant modification within the SN limbs after the first 5 days (Fig. 1G).

### 3.2 SN-related changes in cortical bone exhibit slower onset yet faster stabilization

**Fig. 2:**
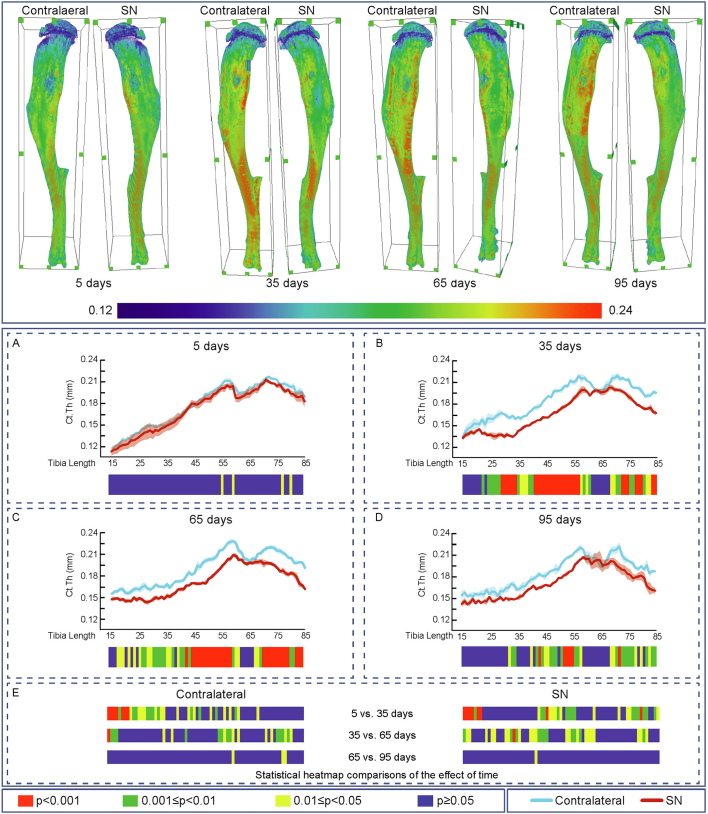
Cortical mean thickness decreased at 35 days of Sciatic neurectomy. A: Cortical thickness heat map. Mean ± Standard error of Mean thickness (Ct.Th) and statistical heat maps of 12 week-old-mice left tibia (Contralateral) and right tibia (Sciatic Neurectomy-SN) after B: 5 (*n* = 5), C: 35 (*n* = 4), D: 65 (*n* = 5) and E: 95 (*n* = 3) days of SN, F: levels of statistical significance of Contralateral and SN along time. Levels of statistical significance set at *P* < 0.001 (red), 0.001 < *P* < 0.01 (green) and 0.01 < *P* < 0.05 (yellow); *P* > 0.05 (blue).

**Fig. 3:**
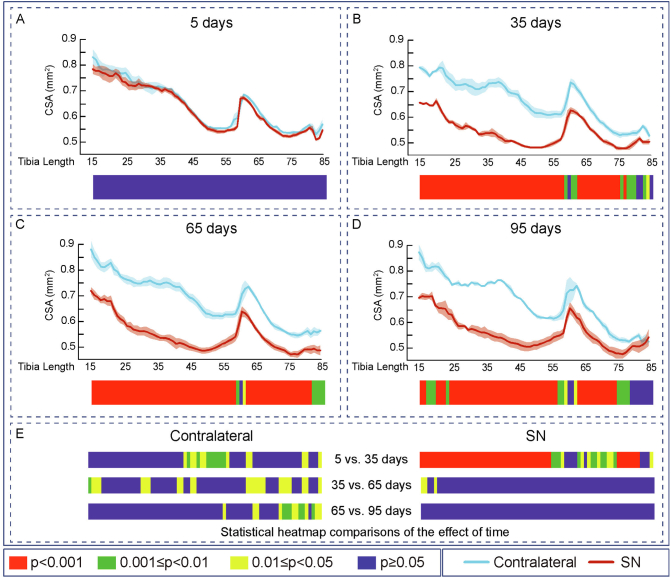
Cortical cross-sectional area decreased and saturated at 35 days of Sciatic neurectomy. Mean ± Standard error of Cross sectional area (CSA) and statistical heat maps of 12 week-old-mice left tibia (Contralateral) and right tibia (Sciatic Neurectomy-SN) after A: 5 (*n* = 5), B: 35 (*n* = 4), C: 65 (*n* = 5) and D: 95 (*n* = 3) days of SN. E: levels of statistical significance of Contralateral and SN along time. Levels of statistical significance set at *P* < 0.001 (red), 0.001 < *P* < 0.01 (green) and 0.01 < *P* < 0.05 (yellow); *P* > 0.05 (blue).

**Fig. 4:**
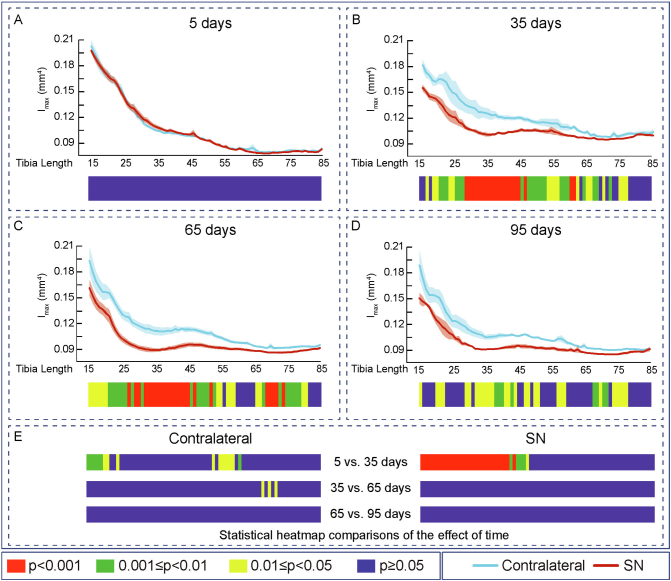
Cortical second moment of area around minor axes decreased and saturated at 35 days of Sciatic neurectomy. Mean ± Standard error of Second moment of area around minor axes (I_max_) and statistical heat maps of 12 week-old-mice left tibia (Contralateral) and right tibia (Sciatic Neurectomy-SN) after A: 5 (*n* = 5), B: 35 (*n* = 4), C: 65 (n = 5) and D: 95 (*n* = 3) days of SN. E: levels of statistical significance of Contralateral and SN along time. Levels of statistical significance set at *P* < 0.001 (red), 0.001 < *P* < 0.01 (green) and 0.01 < *P* < 0.05 (yellow); *P* > 0.05 (blue).

**Fig. 5:**
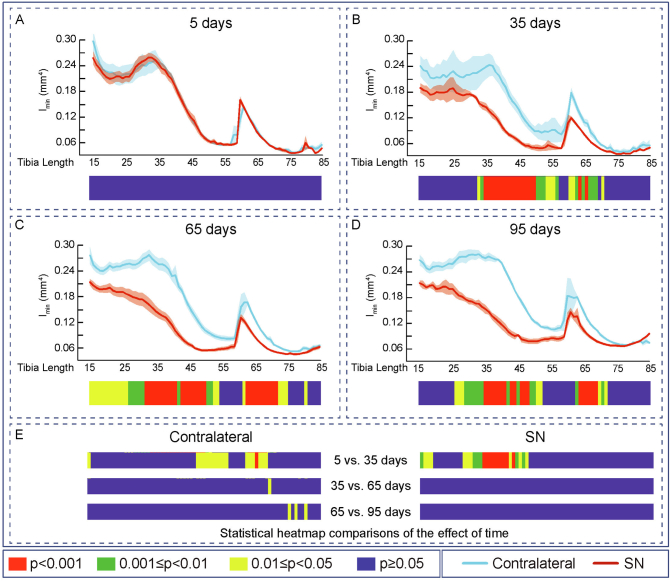
Cortical second moment of area around major axes decreased and saturated at 35 days of Sciatic neurectomy. Mean ± Standard error of Second moment of area around major axes (I_min_) and statistical heat maps of 12 week-old-mice left tibia (Contralateral) and right tibia (Sciatic Neurectomy-SN) after A: 5 (*n* = 5), B: 35 (*n* = 4), C: 65 (n = 5) and D: 95 (*n* = 3) days of SN. E: levels of statistical significance of Contralateral and SN along time. Levels of statistical significance set at *P* < 0.001 (red), 0.001 < *P* < 0.01 (green) and 0.01 < *P* < 0.05 (yellow); *P* > 0.05 (blue).

**Fig. 6:**
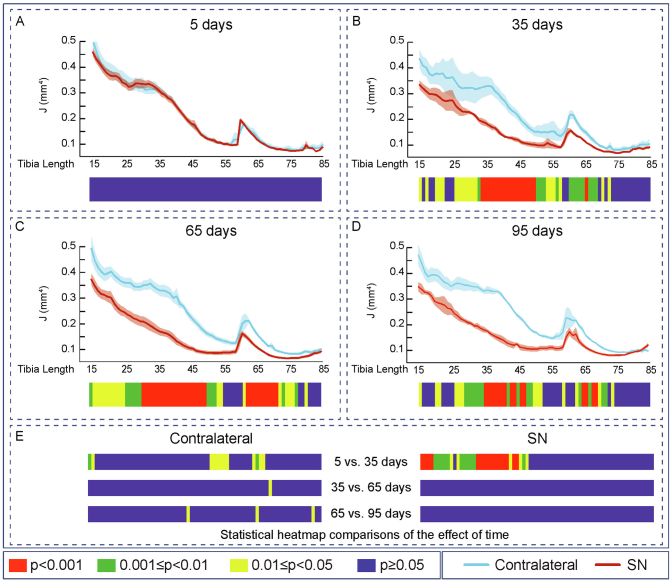
Cortical predict resistant to torsion decreased and saturated at 35 days of Sciatic neurectomy. Mean ± Standard error of Predict resistant to torsion (J) and statistical heat maps of 12 week-old-mice left tibia (Contralateral) and right tibia (Sciatic Neurectomy-SN) after A: 5 (n = 5), B: 35 (n = 4), C: 65 (n = 5) and D: 95 (n = 3) days of SN. E: levels of statistical significance of Contralateral and SN along time. Levels of statistical significance set at P < 0.001 (red), 0.001 < P < 0.01 (green) and 0.01 < P < 0.05 (yellow); P > 0.05 (blue).

**Fig. 7:**
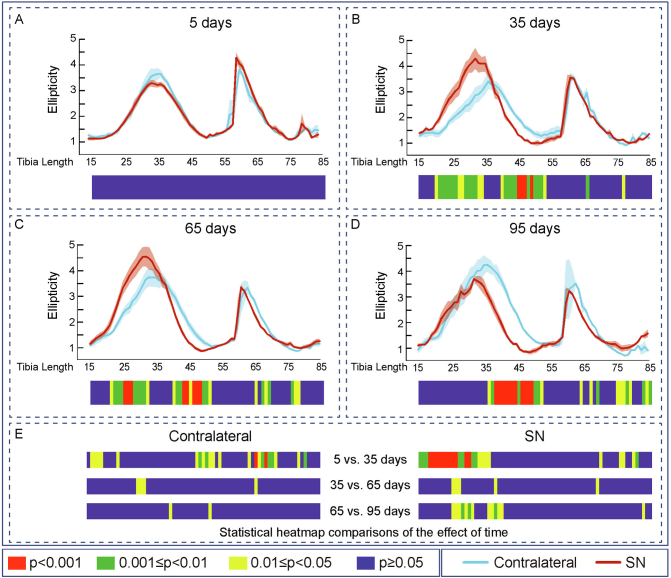
Cortical ellipticity decreased at 35 days of Sciatic neurectomy. Mean ± Standard error of Ellipticity and statistical heat maps of 12 week-old-mice left tibia (Contralateral) and right tibia (Sciatic Neurectomy-SN) after A: 5 (n = 5), B: 35 (n = 4), C: 65 (n = 5) and D: 95 (n = 3) days of SN. E: levels of statistical significance of Contralateral and SN along time. Levels of statistical significance set at P < 0.001 (red), 0.001 < P < 0.01 (green) and 0.01 < P < 0.05 (yellow); P > 0.05 (blue).

**Fig. 8:**
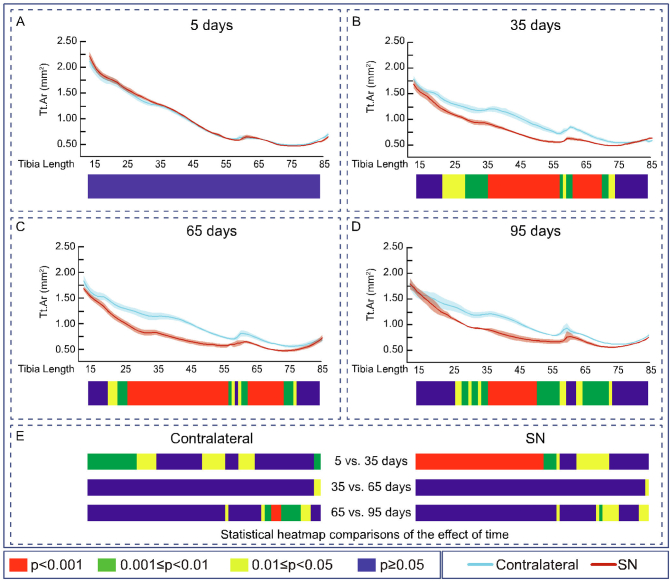
Total tissue area decreased at 35 days of Sciatic neurectomy. Mean ± Standard error of Total tissue area and statistical heat maps of 12 week-old-mice left tibia (Contralateral) and right tibia (Sciatic Neurectomy-SN) after A: 5 (n = 5), B: 35 (n = 4), C: 65 (n = 5) and D: 95 (n = 3) days of SN. E: levels of statistical significance of Contralateral and SN along time. Levels of statistical significance set at P < 0.001 (red), 0.001 < P < 0.01 (green) and 0.01 < P < 0.05 (yellow); P > 0.05 (blue).

Evaluation of the effects of SN showed that cortical thickness (Ct.Th, Fig. 2), cross-sectional area (CSA), I_min_, I_max_, predicted resistance to torsion (J), ellipticity and tissue Area (Tt.Ar) were all unmodified along the extensive examined portion of the tibia during the initial 5 days (Figs. 2A, 3A, 4A, 5A, 6A, 7A and 8A). In contrast, very marked and statistically significant modifications in cortical bone mass and shape were found 35 days after SN, with significant diminution in Ct.Th, CSA, I_min_, I_max_ J and Tt.Ar along almost the entire length of the cortical compartment (Figs. 2B, 3B, 4B, 5B, 6B and 8B). These SN-related changes between 5 and 35 days were accompanied by marked shifts in tibial shape with significant changes in ellipticity (vs. contralateral) between 20 and 40% and 40-55% of the tibia length (Fig. 7B).

Similar patterns of SN-induced modification in Ct.Th, CSA, I_min_, I_max_ J and Tt.Ar in the tibia were apparent at both 65 and 95 days after SN (Fig. 2, Fig. 3, Fig. 4, Fig. 5, Fig. 6, Fig. 8, C and D). Closer interrogation and comparison across these different timepoints within SN limbs revealed that significant modifications were, by contrast to the trabecular bone, almost entirely absent between the 35-65 and 65-95 day-long periods for Ct.Th, CSA, Imin, Imax, J and Tt.Ar (Figs. 2E, 3E, 4E, 5E, 6E and 8E). Cortical ellipticity showed some sporadic shifts during these later timepoints (Fig. 7E). SN did not modify the tissue mineral density (TMD; vs. contralateral) significantly at 5, 35, 65 nor 95 days post-SN.

Comparison across different timepoints within the contralateral limbs disclosed some regionalised, but much less widespread increases in Ct.Th, CSA, I_min_, I_max_, J and ellipticity which were most marked between 5 and 35 days (Fig. 2, Fig. 3, Fig. 4, Fig. 5, Fig. 6, Fig. 7, E in all). The Tt.Ar showed a more marked decrease in the first third of the tibia length between 5 and 35 days (Fig. 8, E). These modifications in bone mass and shape were somewhat prolonged yet were less evident into the 35-65 and 65-95 day period and were mostly localised to the tibia mid-shaft region (Fig. 2, Fig. 3, Fig. 4, Fig. 5, Fig. 6, Fig. 7, Fig. 8, E in all). Together, these data indicate that the response of the cortical compartment to SN was, overall, slower in onset, requiring 35 days, but yet was faster to become stabilized (little change after 35 days) than the corresponding changes in the trabecular architecture within the same bone (see Fig. 1).

## 4 Discussion

Our study establishes that the trajectory of bone loss differs markedly in discrete tibial compartments following long-term SN. We have shown that bone in trabecular regions is more sensitive than the cortical compartment to the rapid effects of SN-related reduced loading, and that the duration of the response to SN in the trabecular bone region is highly protracted when compared to the cortical compartment in which these somewhat delayed SN-related modifications in mass and architecture are more promptly stabilized. SN-induced tibial trabecular bone loss thus appears to exhibit faster onset and to require more time to plateau than cortical bone, showing marked changes in mass and architecture within 5 days that continue until at least day 65 post-SN. These trabecular modifications include a diminution in trabecular bone volume (BV/TV), trabecular number (Tb.N), trabecular thickness (Tb.Th) and bone mineral density (BMD). On the other hand, cortical bone mass and shape (Ct.Th, CSA, I_min_, I_max_ and J) did not show any evidence of deterioration until 35 days post-SN, with no marked changes in these parameters thereafter; the architectural SN-related cortical change apparent later (35-95 days) were tissue area and ellipticity. These data support divergent responses to SN in trabecular and cortical bone compartments within a single bone.

Botulinum- or SN-induced reduction in loading of bones has been reported not to modify their overall length (Weinreb et al., 1989; Bouvard et al., 2012). Our data are consistent with these observations, as we find that tibial length appears not to be dissimilar between contralateral limbs and limbs subjected to prolonged SN. This is despite some divergence between left and right limbs, with left contralateral limb tibiae showing increased length between 35 and 65 days whilst longitudinal growth of tibiae in limbs subjected to right side SN failed however to reach statistical significance. This suggests that SN does not dramatically influence longitudinal growth and that this modest effect is highly unlikely to impact our analyses at matched anatomical sites/ROI within the tibia. The general trend for increased length agrees with expected limb bone growth kinetics as the 12 week-old mice at the beginning of the experiment age to 25-26 weeks across the duration of the experimental phase and with the attainment of skeletal maturity with peak bone mass at ~22 weeks which coincides with the 65 day post-SN time-point (Javaheri et al., 2019).

Rapid trabecular resorption is a well-established hallmark of reduced mechanical loading. It is considered a product of a ‘triggering’ of the mechanostat, which in turn is responsible for prompting increases in resorption and decreases in formation in the absence of habitual levels of active load bearing (Weinreb et al., 1989; Frost, 1987; Otzel et al., 2019; Biewener and Bertram, 1994; Piet et al., 2019). These increases in resorption in response to reduced loading can be particularly rapid. Earlier studies have described activated expression of pro-osteoclastic inflammatory genes and later of osteoclasts within the bone marrow after only 1-3 days of reduced loading induced by botulinum toxin injection (Ausk et al., 2017). These inflammatory-related processes are considered responsible for the extensive, acute phase degradation of trabeculae in response to imposed unloading/disuse. The raised sensitivity of the trabecular bone to unloading-related degradation has been described previously through comparison with cortical bone (Otzel et al., 2019; Yang et al., 2019). Our studies align with these observations by showing that bone in trabecular region of the tibia is more sensitive than the tibial cortices to the rapid effects of SN-related reduction in loading. The decreases in trabecular BV/TV, number, thickness and BMD that occur in the limbs subjected to 5 days of SN are further demonstration of osteoclast-mediated, particularly targeted, demise of bone in this compartment.

Our data also showed that the SN-induced divergence in trabecular BV/TV, number and BMD was either maintained or became more pronounced after 35, 65 and 95 days. It was somewhat surprising, however, that trabecular thickness did not follow this same trend. Interrogation and closer integration with data showing that trabecular number in contralateral limbs is consistently greater than in tibiae subjected to SN at all time-points, prompts speculation that these shifts in *mean* trabecular thickness are likely the product of the complete loss of all thin trabeculae in the relatively under-loaded SN tibiae. It is tempting to speculate that this due to an aggressive early SN-induced resorption, which effectively increases the *mean* thickness of the remaining trabeculae to those levels seen in contralateral, internal control tibiae. This interpretation that SN targets the thinner trabeculae for aggressive resorption is indeed strengthened by measures of connectivity density which are lower in the SN limb (vs. contralateral) at 35 days. These findings are similar to those reported by Otzel et al. (2019) in rats and emphasise the importance of always evaluating trabecular number, thickness and connectivity density in order to avoid potential data misinterpretation. They also pinpoint the interesting possibility that thinner trabeculae are more targeted for resorption during the early phases of SN-induced decreased loading of the tibia and raise questions regarding the underpinning mechanisms.

The changes in trabecular architecture at 35 and 65 days post-SN are more difficult to interpret. They indicate an ongoing demise of trabecular BV/TV and number at 5-35 days post-SN that may be due to the combined effects of enhanced resorption and diminished formation in the trabecular compartment. They also imply that if these responses are indeed mechanostat-driven, that they are somewhat protracted. This notion is supported by data at 65 days, which indicate an ongoing and significant further decline in BV/TV and trabecular number during this late phase of SN; these SN-related trabecular changes indeed only cease in the latest 65-95 day period. It is important to stress that the 35 day time-point does, however, represent somewhat of a watershed in trabecular architecture in the contralateral tibia, when it seems that indices of trabecular mass appear to peak. Examination of trabecular BV/TV, number and BMD reveals that they both decline, without any change in Tb.TMD, between days 35 and 65 (mice ~4-5 months old) in the contralateral limbs, indicative of a potential age-related decline in the trabecular compartment (Glatt et al., 2007). The fact that SN generates more marked declines in the trabecular compartment, which are reduced relative to the contralateral limb even into the later 65-95 day period (mice ~5-6 months old) serve only to emphasise the prolongation of the SN-induced changes (De Souza et al., 2017; Glatt et al., 2007). How or why these changes are protracted remains to be fully established.

BMD is a critical parameter in the avoidance of bone fracture (Dauty et al., 2000; Keating et al., 1992). In humans, BMD decreases by ~40% in the first year after a spinal cord-injury (Dauty et al., 2000). Herein, we demonstrate that trabecular BMD diminishes by 33, 49, 51 and 44% at 5, 35, 65 and 95 days after SN, respectively, compared to respective contralateral limbs. Kingery et al. (2003) had previously reported decreased BMD in the contralateral limb of rats subjected to SN, which they found to be further exacerbated by 2-weeks of daily substance P receptor antagonist application; this was interpreted to indicate a role of Substance P signaling in bone loss (Kingery et al., 2003). Our work also demonstrates decreased trabecular BV/TV, number and BMD in contralateral limbs in a similar mouse model of SN that could be similarly indicative of contralateral neuronal signaling and a systemic response to local injury (Kingery et al., 2003).

In clear contrast to the trabecular compartment, it is readily apparent from our data that cortical bone in the tibia is less sensitive to the rapid effects of SN; with SN-related effects taking up to 35 days. This contrast extends to the duration of the SN effect, which in all cortical tibia regions is much less protracted and more promptly stabilized compared to trabecular bone. These changes are nonetheless consistent with the triggering by reduced loading of a negatively balanced mechanostat, almost across the entire cortical compartment (Weinreb et al., 1989; Frost, 1987; Otzel et al., 2019; Biewener and Bertram, 1994; Ausk et al., 2017). It is important to point out that the trajectory of the SN-induced cortical changes we observe, suggest that they either have a somewhat slower onset of activation or that their impact plays out more slowly in the cortices (taking 35 days rather than 5 days to emerge). It is therefore tempting to merge these observations and to speculate that the initial phase of SN-related reduced loading preferentially targets trabecular bone and only thereafter are changes in the cortical compartment initiated.

Our whole bone analysis shows that upon the emergence of these SN-related cortical mass and shape changes, their scale is nonetheless incredibly extensive, with modification in both being very dramatic along almost the entire tibia length. There are regions that intriguingly remain somewhat protected from these changes. We find that the tibiofibular junction (identified by the ‘peak’ in the graphs at between 60 and 65% of the total tibia length) is one such region where cortical bone loss is considerably lower. The reasons for this apparent protection remain somewhat mysterious, but they might be related to the privilege attributed to this bony *superstructure* during extensive longitudinal expansion of the tibia; previous studies have established that the tibia *scales* the rates of proximal and distal elongation to retain the junction in a relatively unshifting position during periods of tibial growth (Stern et al., 2015; Javaheri et al., 2020).

Our data show that Ct.Th, CSA, I_min_, I_max_, J ellipticity, and Tt.Ar are all also more marked in the proximal tibial regions and that some of the most distal tibial regions are relatively devoid of SN-induced changes in these indices of bone mass and shape. This resembles data from studies that have focused on long-term adaptation in tibia shape in response to supra-physiological strains applied non-invasively in a mouse bone-loading model, which discovered long-term increases in tibial curvature that are consistent with an attempt to adjust load predictability in the whole tibia in a manner that appears independent of local strain. It is tempting to speculate that these long-term load-induced shifts in tibial shape that become evident only after 100 days engage similar mechanisms as those employed to achieve the chronic changes on ellipticity we describe herein, after 95 days of SN (Javaheri et al., 2020).

Bone shape is indeed a good strength predictor because it allows the bone to resist fracture-inducing strain levels at the local and organ level (Biewener and Bertram, 1994; Javaheri et al., 2020). Like indices of bone mass, cortical bone shape (I_min_, I_max_ and J) showed major changes only 35 days of SN and these did not exhibit any marked modification thereafter. This markedly speedier stabilization of cortical bone shape has to our knowledge not been reported upon previously; its basis is unknown but its promptness suggests that this is the ultimate target of the (re)modelling that has taken place in response to the diminished loading linked to SN and, that once attained it remains unmodified. It is interesting that the pattern of ellipticity changes does not track to other indices of bone shape and that this particular feature instead continues to respond for some considerable time (through to 95 days) in response to the imposition of SN. These observations point to a divergence in the trajectory of the tibial cortical response to SN-related reduced loading, with changes in mass preceding the modelling of overall tibial *organ* shape thereafter. However, our findings also indicate that the final organisation of the tibial cortex 95 days after SN assumes a somewhat straighter overall shape than the more curved contralateral tibia. This has previously been found to imperil bones to potential bending in unpredicted directions in response to load, thus jeopardizing structural integrity and increasing fracture risk predisposition (Biewener and Bertram, 1994; Javaheri et al., 2020).

Another feature that contributes to fracture risk predisposition is tissue mineral density (TMD). Our findings showed that SN did not evoke any modification in cortical nor trabecular TMD, relative to the contralateral limb, during any of four times points examined. An elegant study using similar 3D analyses with phantom calibration has, however, reported site-specific shifts in femoral TMD in response to spinal cord injury in rats, within the diaphyseal and not the distal epiphyseal region (Otzel et al., 2019); the basis for this discrepancy in site-specific shifts in cortical tissue mineral density in the two studies is not clear.

There are several caveats that apply to our data. For example, we cannot assume that the limb subjected to SN necessarily experiences total isolation from mechanical load bearing. It has indeed been shown that such rodent models show only a reduction of 27% of maximum ipsilateral hind paw weight-bearing post-SN (Kingery et al., 2003). It is also salient to be cognisant that mice use tetrapedal locomotion and are readily capable of asymmetrically distributing their weight to both contralateral hind and both fore-paws in response to SN. The complexity of this redistribution of weight-bearing during locomotion is emphasised by the studies of Kingery et al. (2003) which have shown a lack of any significant shift in contralateral limb weight-bearing following SN in rats. The lack of control limbs from otherwise completely untreated mice precludes specific adjustment for any systemic modifications that may have exerted effects upon the contralateral limb, or which may have interacted to generate divergent age-related changes in bone mass and architecture that are known to occur, for instance, in the trabecular bone of female C57BL/6 J between 8 and 24 weeks of age (Glatt et al., 2007). It remains possible, therefore, that the SN-induced changes that we have observed may differ in mice that are either younger and still growing, or older and having reached skeletal maturity prior to imposition of SN. Elegant studies exploring the effects of either applied mechanical load or relative unloading (by a range of strategies) using control limbs from otherwise untreated mice to exclude any such systematic effects have, nonetheless, shown similar findings to ours; this is reinforced by further studies, such as those imposing botulinum toxin injection, in which internal contralateral controls have been employed for comparison (Otzel et al., 2019; Jiang et al., 2006; Lodberg et al., 2015; Piet et al., 2019; Kingery et al., 2003; Javaheri et al., 2020). Another caveat concerns the possible Type I error that may emerge due to the number of animals used, which is smaller than in other similar studies, and thus it is important to highlight the absence of any outliers (ROUT method), that all data passed normality tests and provided sufficient power with the sample size we have used (Mascha and Vetter, 2018). Furthermore, the absence of any indices of dynamic bone remodelling from our work make meaningful tracking of the trajectory of bone resorption and (re)modelling activity impossible. This makes it clear that future studies exploring the effects of SN would be significantly enriched through the use of in-vivo microCT in order to spatially track disuse-induced bone shape changes within an individual bone with time (Piet et al., 2019; Javaheri et al., 2020).

There are also two points that will clearly demand future clarification. Firstly, why it is that cortical and trabecular bone behave differently? Possible explanations may consider findings indicating that the micro-architectural changes in bone mass that occur in response to applied load at the midshaft cortical bone, but not those in proximal cancellous bone, are necessarily related directly to strain levels (Yang et al., 2019). Similarly, they may also accommodate observations indicating that trabecular and cortical bone exhibit divergent load-induced behaviours after the imposition of prolonged periods of disuse. Thus, cortical but not trabecular bone load responses are rescued by the imposition of SN (over both short and long time frames) and by tail suspension, suggesting that reduced history of loading may emphasise differences in mechanoadaptive response in these two bone compartments (De Souza et al., 2017; Yang et al., 2019; Javaheri et al., 2020). Secondly, why are some cortical regions more markedly affected than others? In conclusion, our data from maturing adult female mice indicate that trabecular and cortical compartments of the tibia behave modularly in response to SN. This modular trabecular behaviour is characterised by a more protracted duration of response and greater sensitivity to the rapid effects of SN, whilst cortical bone instead exhibits a somewhat delayed onset but more rapidly stabilized set of SN-related modifications in mass and architecture, where only overall *organ* shape change are a later yet ultimate target.

## CRediT authorship contribution statement

Designed the experiment: BJ, RLS and AAP; Executed the experiment: RLS; Scanned the bones: BJ; Analysed the images: SM and BJ; Analysed the data: SM, BJ and AAP; Made the figures: SM and BJ; Wrote the first draft of the manuscript: SM and AAP; Wrote and approved the final version of manuscript: SM, BJ, RLS and AAP.

## Declaration of competing interest

None.

## Appendix A Supplementary data

Supplementary data Mean ± SEM of trabecular tissue mineral density (Tb.TMD) of 12 week-old-mice left tibia (Contralateral) and right tibia (Sciatic Neurectomy-SN) after 5, 35, 65 and 95 days of SN. Statistical significance: * *p* < 0.05 when compare between groups (contralateral vs SN). $: p < 0.05 when compare 5 vs 35 days inside same group (contralateral vs contralateral or SN vs SN). #: p < 0.05 when compare 35 vs 65 days inside same group (contralateral vs contralateral or SN vs SN). Supplementary data
