# Supplementary material for: Sciatic neurectomy-related cortical bone loss exhibits delayed onset yet stabilises more rapidly than trabecular bone
Source: Bone Rep. 2021 Aug 17;15:101116. doi: 10.1016/j.bonr.2021.101116 (PMC8387754; doi:10.1016/j.bonr.2021.101116)
Supplement: Supplementary data — Mean ± SEM of trabecular tissue mineral density (Tb.TMD) of 12 week-old-mice left tibia (Contralateral) and right tibia (Sciatic Neurectomy-SN) after 5, 35, 65 and 95 days of SN. Statistical significance: * p < 0.05 when compare between groups (contralateral vs SN). $: p < 0.05 when compare 5 vs 35 days inside same group (contralateral vs contralateral or SN vs SN). #: p < 0.05 when compare 35 vs 65 days inside same group (contralateral vs contralateral or SN vs SN). [file mmc1.docx]

**
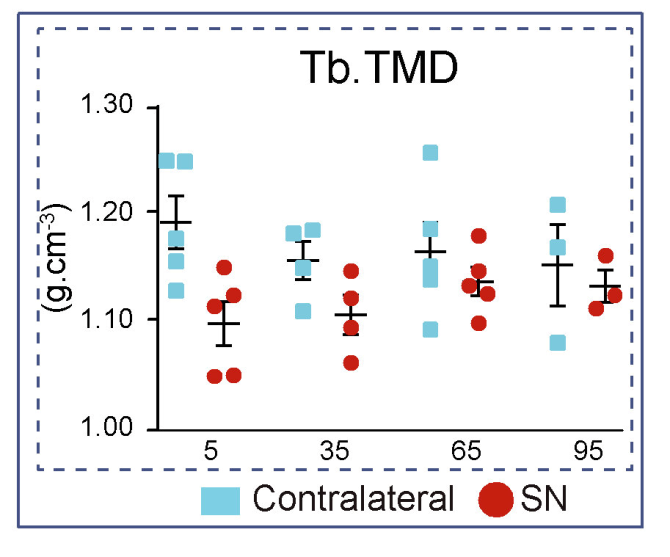
**

**Supplementary data.** Mean ± SEM of trabecular tissue mineral density (Tb.TMD) of 12 week-old-mice left tibia (Contralateral) and right tibia (Sciatic Neurectomy-SN) after 5, 35, 65 and 95 days of SN. Statistical significance: * *p*<0.05 when compare between groups (contralateral vs SN). $: p < 0.05 when compare 5 vs 35 days inside same group (contralateral vs contralateral or SN vs SN). #: p < 0.05 when compare 35 vs 65 days inside same group (contralateral vs contralateral or SN vs SN).
